# The relationship between hemoglobin and triglycerides in moyamoya disease: A cross-sectional study

**DOI:** 10.3389/fneur.2022.994341

**Published:** 2022-09-08

**Authors:** Yu Su, Genhua Li, Huihui Zhao, Song Feng, Yan Lu, Jilan Liu, Chao Chen, Feng Jin

**Affiliations:** ^1^Clinical Medical College, Jining Medical University, Jining, China; ^2^Department of Neurosurgery, Affiliated Hospital of Jining Medical University & Shandong Provincial Key Laboratory of Stem Cells and Neuro-Oncology, Jining, China; ^3^Medical Research Center, Affiliated Hospital of Jining Medical University, Jining Medical University, Jining, China

**Keywords:** moyamoya disease, internal carotid arteries, cerebrovascular disease, triglycerides, hemoglobin

## Abstract

Hemoglobin (Hb) and lipid metabolism are critical in the pathophysiology of moyamoya disease (MMD), and Hb and triglycerides (TGs) both play roles in the development of cerebrovascular illness. However, there is little evidence of a link between Hb and TGs in patients with MMD. This study aimed to determine the association between Hb and TGs in patients who had recently been diagnosed with MMD. From March 2013 to December 2018, 337 patients clinically diagnosed with MMD were admitted to our hospital. Among these, 235 were selected for analysis in this retrospective, cross-sectional study. Each patient's clinical features were documented. For analysis, we used univariate analysis, smoothed-curve fitting, and multivariable, piecewise linear regression. Overall, the mean±standard deviation patient age was 48.14 ± 11.24 years, 44.68% were men, and the mean Hb concentration was 135.72 ± 18.99 g/L. After controlling for relevant confounders, smoothed-curve fitting revealed a nonlinear association between the Hb and TG concentrations (*P* = 0.0448). When the Hb concentration was below 141 g/L, multivariate piecewise linear regression analysis revealed a significant association between the Hb and TG concentrations [β: 0.01, 95% confidence interval (CI): 0.00, 0.01; *P* = 0.0182], although the association disappeared above this threshold (β:−0.00, 95% CI:−0.01, 0.01; *P* = 0.4429). In individuals newly diagnosed with MMD, there is a significant correlation between Hb and TGs, which may be connected to MMD pathogenesis.

## Introduction

Moyamoya disease (MMD) is a chronically occlusive cerebrovascular illness marked by stenosis of the terminal portions of both internal carotid arteries (ICAs), which leads to the creation of an aberrant network of collateral vessels. Its progressive phase can often lead to ischemic and hemorrhagic strokes (1–3). Thus, given the high risk of stroke in patients with MMD, it is particularly important to prevent the occurrence and development of this disease. However, despite more than 60 years of continuous research, its mechanisms remain unclear. Recent research suggests that TGs is a risk factor for cerebrovascular diseases, including carotid artery stenosis and intracranial artery stenosis (4, 5). Therefore, exploring the factors associated with abnormal lipid metabolism could facilitate our knowledge of the its fundamental mechanics. In a previous study, we discovered that uric acid and triglycerides (TGs) had a substantial beneficial relationship, and the early prevention of hyperuricemia and lipid abnormalities was associated with a decrease in the incidence of MMD (6).

Hb is a cellular protein that binds to oxygen (7, 8). There is a positive association between the Hb concentration and inflammation in previously and currently infected populations (7, 9, 10). Furthermore, the Hb concentration is a well-known independent risk factor for stroke and a poor prognosis (11, 12). In general, both Hb and TGs play crucial roles in the pathology and physiology of cerebrovascular disease, although the relationship between the two has not been elucidated.

Taken together, MMD is a cerebrovascular disease that frequently leads to a stroke, while Hb and TGs are both linked to the development of a stroke. Therefore, this study aimed to investigate whether Hb and TGs are independently associated with each other among patients newly diagnosed with MMD in China. Clarifying the relationship between the two may help to predict the type of stroke induced by MMD and to better understand their role the development of MMD.

## Methods

### Study design

The link between Hb and TGs was studied using a retrospective, cross-sectional design. The Hb concentration was defined as the independent variable and the TG concentration as the predictor variable.

### Study population

All data used in this study were acquired from the computerized medical record system of the Affiliated Hospital of Jining Medical University. The information we gathered did not contain any personal information, to protect patient privacy. Informed consent was not required because this cohort study was retrospective. The hospital's institutional review board approved this study.

Data of 235 patients were initially collected. The start and end dates for inclusion were March 2013 and December 2018, respectively. The MMD (Spontaneous Occlusion of the Circle of Willis) Guidelines for Diagnosis and Treatment (2012 Edition) were used to guide the clinical strategy for each participant (13). The following cerebral angiography results were required for diagnosis: (1) in the arterial phase, stenosis or blockage of the intracranial ICA, parietal part of the anterior cerebral artery, and/or middle cerebral artery; (2) anomalies in vascularization next to an endothelial orstenotic lesion in the first cycle; and (3) observations in (1) and (3) must be consistent with those in (2). The following were the exclusion criteria: (1) short-term use of anemia-correcting drugs, (2) atherosclerosis, (3) autoimmune disease, (4) meningitis, (5) brain tumors, (6) Down syndrome, (7) cerebrovascular lesions detected via head irradiation, (8) head injury, (9) von Recklinghausen's disease, (10) age <18 years and (11) other conditions. We only included patients newly diagnosed with MMD who were admitted to our hospital. Further exclusion criteria were patients with myeloproliferative illnesses receiving toxic treatments, pregnant women, breastfeeding women, patients currently using diuretics or lipid-regulating medicines, patients with renal or liver illness, and patients on antinociceptive drugs.

### Variables

Hb and TG concentrations were measured at the start of the study and used as continuous variables. Briefly, the department nurse collected the patients' peripheral venous blood while they were fasting, instantly submitting it to the laboratory. All measurements were performed at our hospital laboratory by laboratory technicians and physicians.

The covariates in this investigation were divided into three categories: (1) demographic data; (2) variables previously reported to affect the Hb and/or TG concentration; and (3) variables identified based on our clinical experience. As a result, multivariable models were constructed, adjusting for the following: (1) quantitative variables: sex, smoking status, and alcohol intake and (2) continuous variables: age and body mass index (BMI). All these variables were obtained at baseline.

### Equations

Continuous variables are displayed as means ± standard deviations, and categorical variables are displayed as frequencies and percentages. All analyses were performed using the R statistical package (http://www.R-project.org, R Foundation for Statistical Computing, Vienna, Austria) and EmpowerStats (http://www.empowerstats.com, X&Y Solutions, Inc., Boston, MA). Statistical significance was defined as a *P* < 0.05 (two-tailed). For details of the statistical methods, please see the Supplementary material.

## Results

### Clinical features

**Figure 1:**
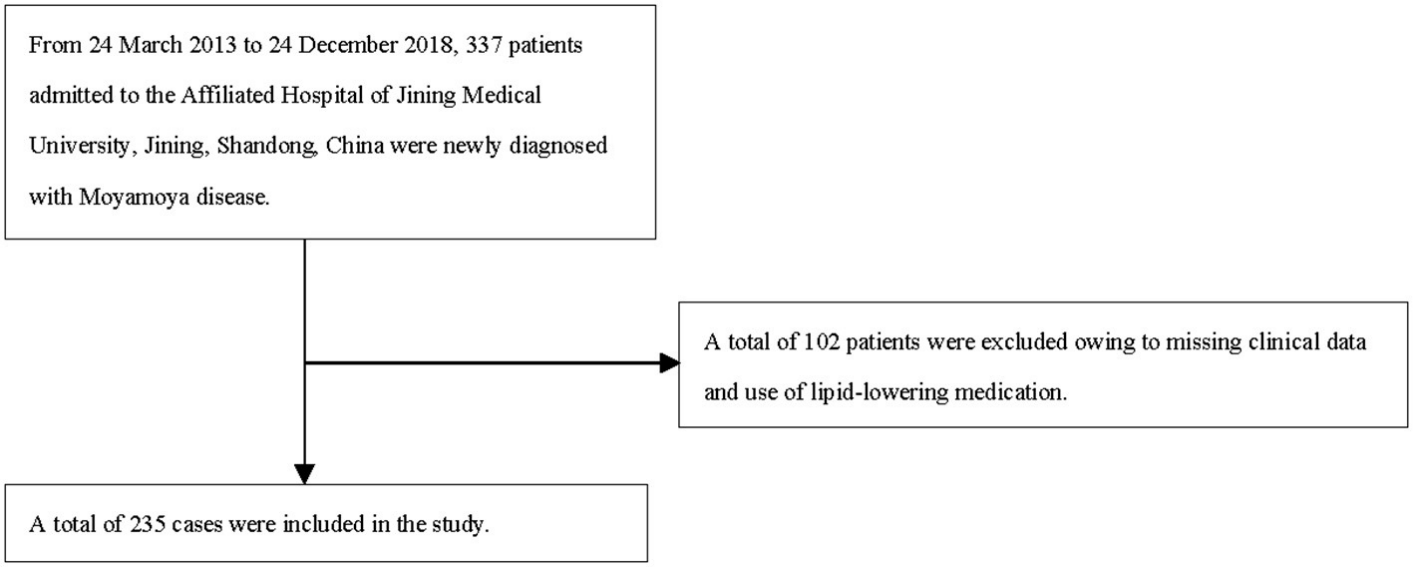
Flowchart of inclusion and exclusion criteria.

We included 235 patients were identified for the final data analysis (Figure 1). They had an average age of 48.14 ± 11.24 years; 44.68% were men, and 27.75% had hemorrhagic MMD (Table 1). The TG and Hb concentrations were 1.30 ± 0.82 mmol/L and 135.72 ± 18.99 g/L, respectively.

**Table 1:** Clinical characteristics of the study population.

**Variable**	**Total**
Number of cases, *n*	235
Age (years, mean ± SD)	48.14 ± 11.24
BMI (kg/m^2^, mean ± SD)	25.42 ± 3.47
**Sex**, ***n*** **(%)**	
Male	105 (44.68)
Female	130 (55.32)
**Current smoker**, ***n*** **(%)**	
No	169 (71.91)
Yes	66 (28.09)
**Alcohol consumption**, ***n*** **(%)**	
No	174 (74.04)
Yes	61 (25.96)
**Disease type**	
Hemorrhagic	58 (27.75)
Ischemic	151 (72.25)
TGs (mmol/L, mean ± SD)	1.30 ± 0.82
TC (mmol/L, mean ± SD)	4.17 ± 0.90
HDL-C (mmol/L, mean ± SD)	1.19 ± 0.23
LDL-C (mmol/L, mean ± SD)	2.42 ± 0.70
VLDL-C (mmol/L, mean ± SD)	0.56 ± 0.36
Lipoprotein (mmol/L, mean ± SD)	265.03 ± 304.83
Hb (g/L)	135.72 ± 18.99

Hb, hemoglobin; TGs, triglycerides; TC, total cholesterol; HDL-C, high-density lipoprotein cholesterol; LDL-C, low-density lipoprotein cholesterol; VLDL-C, very low-density lipoprotein cholesterol.

### Univariate analysis for TGs

Table 2 displays the results of the univariate analyses. Therein, age, sex, smoking status, drinking habits, ischemic disease type, low-density lipoprotein cholesterol concentration, lipoprotein concentration, and Hb concentration were not linked with TG concentration. Furthermore, high-density lipoprotein cholesterol was negatively associated with TG concentration (β: −1.09, 95% confidence interval [CI]: −1.53, −0.65), whereas BMI (β: 0.06, 95% CI: 0.03, 0.09), total cholesterol concentration (β: 0.29, 95% CI: 0.18, 0.40), and very low-density lipoprotein cholesterol (β: 1.75, 95% CI: 1.55, 1.95) were positively associated with TG concentration.

**Table 2:** Univariate analysis for TGs (mmol/L).

**Covariate**	***β*** **(95% CI)**	***P*** **-value**
Age, years	−0.00 (−0.01, 0.01)	0.9422
BMI, kg/m^2^	0.06 (0.03, 0.09)	<0.0001
**Sex**, ***n*** **(%)**		
Male	Reference	
Female	−0.09 (−0.30, 0.12)	0.3854
**Smoking**, ***n*** **(%)**		
No	Reference	
Yes	0.13 (−0.11, 0.36)	0.2851
**Alcohol consumption**, ***n*** **(%)**		
No	Reference	
Yes	0.18 (−0.06, 0.42)	0.1361
**Disease type**, ***n*** **(%)**		
Hemorrhagic	Reference	
Ischemic	0.15 (−0.09, 0.39)	0.2210
TC, mmol/L	0.29 (0.18, 0.40)	<0.0001
HDL-C, mmol/L	−1.09 (−1.53, −0.65)	<0.0001
LDL-C, mmol/L	0.14 (−0.01, 0.29)	0.0618
VLDL-C, mmol/L	1.75 (1.55, 1.95)	<0.0001
Lipoprotein, mmol/L	−0.00 (−0.00, 0.00)	0.9993
Hb, g/L	0.01 (−0.00, 0.01)	0.0628

Hb, hemoglobin; TGs, triglycerides; TC, total cholesterol; HDL-C, high-density lipoprotein cholesterol; LDL-C, low-density lipoprotein cholesterol; VLDL-C, very low-density lipoprotein cholesterol.

### Unadjusted and adjusted linear regression results

Models were created to explore the indirect effects of Hb on the TG concentration after confounders were removed (multivariable linear regression). The effect sizes (*β*) and 95% CIs are shown in Table 3. The model-based effect size in TG concentration in the unadjusted model (Model I) is estimated for a 1 g/L increase in the Hb concentration. For sensitivity analysis, Hb was transformed from a continuous to a categorical variable (quartiles). The *P*-value for the trend in the Hb concentration in the fully adjusted model was similar to the results when Hb concentration was treated as a continuous variable.

**Table 3:** Relationship between Hb (g/L) and TGs (mmol/L) in different models.

**Variable**	**Model I**		**Model II**		**Model III**	
	***β*** **(95% CI)**	***P-*** **value**	***β*** **(95% CI)**	***P*** **-value**	***β*** **(95% CI)**	***P*** **-value**
Hb, g/L	0.01 (−0.00, 0.01)	0.0628	0.00 (−0.00, 0.01)	0.0874	0.00 (0.00, 0.01)	0.0448
**Hb (min-max)**						
Q1 (67–125)	Reference		Reference		Reference	
Q2 (126–136)	0.30 (0.00, 0.59)	0.0512	0.17 (−0.04, 0.39)	0.1106	0.22 (0.03, 0.40)	0.0255
Q3 (137–146)	0.28 (-0.02, 0.58)	0.0641	0.25 (0.03, 0.47)	0.0291	0.24 (0.04, 0.44)	0.0189
Q4 (147–183)	0.30 (0.01, 0.59)	0.0453	0.20 (−0.05, 0.45)	0.1253	0.20 (−0.02, 0.43)	0.0801

Model II adjusted for sex; age; smoking status; alcohol consumption; BMI; disease type; TC; HDL-C; LDL-C; VLDL-C; and lipoproteins.

Model III adjusted for: sex; age (smooth); smoking; alcohol consumption; BMI (smooth); disease type; TC (smooth); HDL-C (smooth); LDL-C (smooth); VLDL-C (smooth); and lipoprotein (smooth).

### Association between Hb and TG concentrations

**Figure 2:**
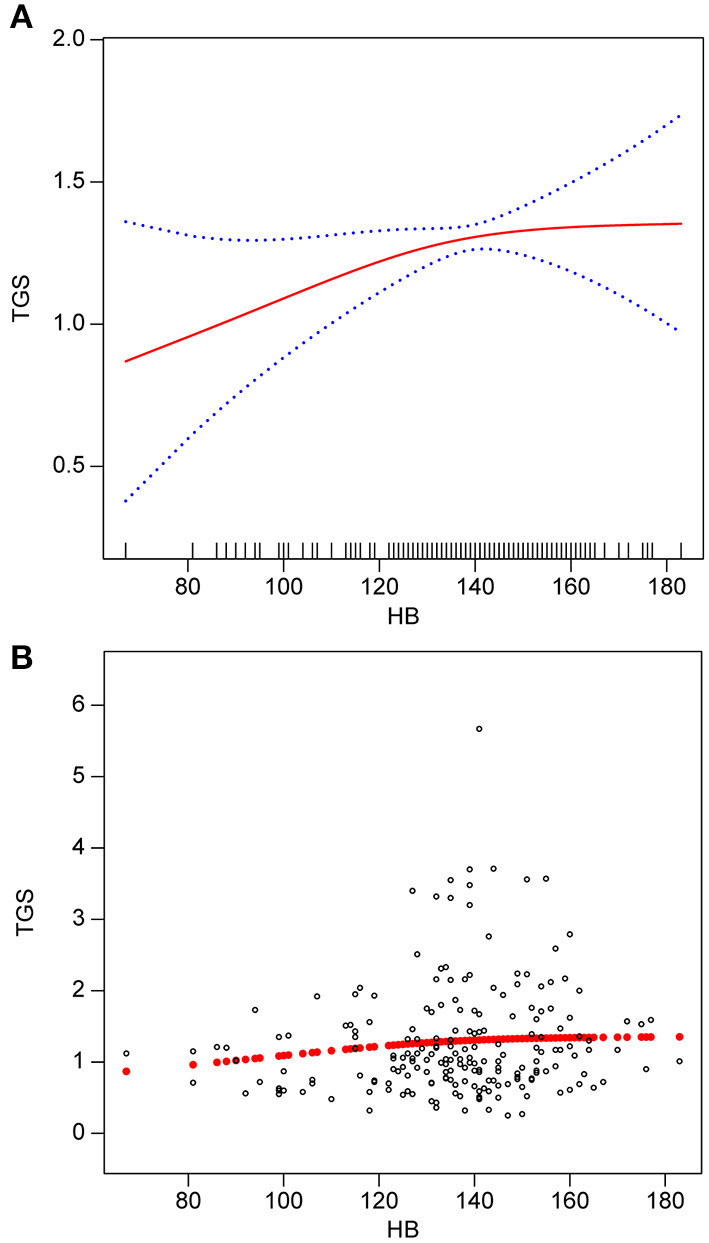
Association between Hb (g/L) and TGs (mmol/L). **(A)** smooth fitted curve of Hb and TGs, **(B)** scatter plot for the distribution of Hb and TGs. The solid red line represents the smooth curve fit between the variables. The blue bands represent the 95% CI of the fit. The model is adjusted for sex; age; smoking status; alcohol consumption; BMI; disease type; TC; HDL-C; LDL-C; VLDL-C; lipoproteins.

Figure 2 illustrates the smooth curve fitting after controlling for possible confounders. The TG concentration had a non-linear relationship with the Hb concentration. The threshold effect was further investigated using curve fitting, as summarized in Table 4. A significant positive correlation between Hb and TG concentrations was identified when the Hb concentration was below 141 g/L (β: 0.01, 95% CI: 0.00, 0.01; *P* = 0.0182). When the Hb concentration was more than 141 g/L, there was no clinically significant link between the two parameters (β: −0.00, 95% CI −0.01, 0.01; *P* = 0.4429).

**Table 4:** Threshold effect analysis of the relationship between Hb and TG levels.

**TGs (mmol/L)**		
	**Adjusted *β* (95% CI)**	***P-*** **value**
**Model I**		
Linear effect	0.00 (−0.00, 0.01)	0.0874
**Model II**		
Inflection point (K)	141	
<141, effect 1	0.01 (0.00, 0.01)	0.0182
>141, effect 2	−0.00 (−0.01, 0.01)	0.4429

Model I, linear analysis; Model II, non-linear analysis. Adjusted variables: sex; age; smoking status; alcohol consumption; BMI; disease type; TC; HDL-C; LDL-C; VLDL-C; and lipoproteins. P < 0.05 was considered statistically significant.

### Subgroup analysis

**Figure 3:**
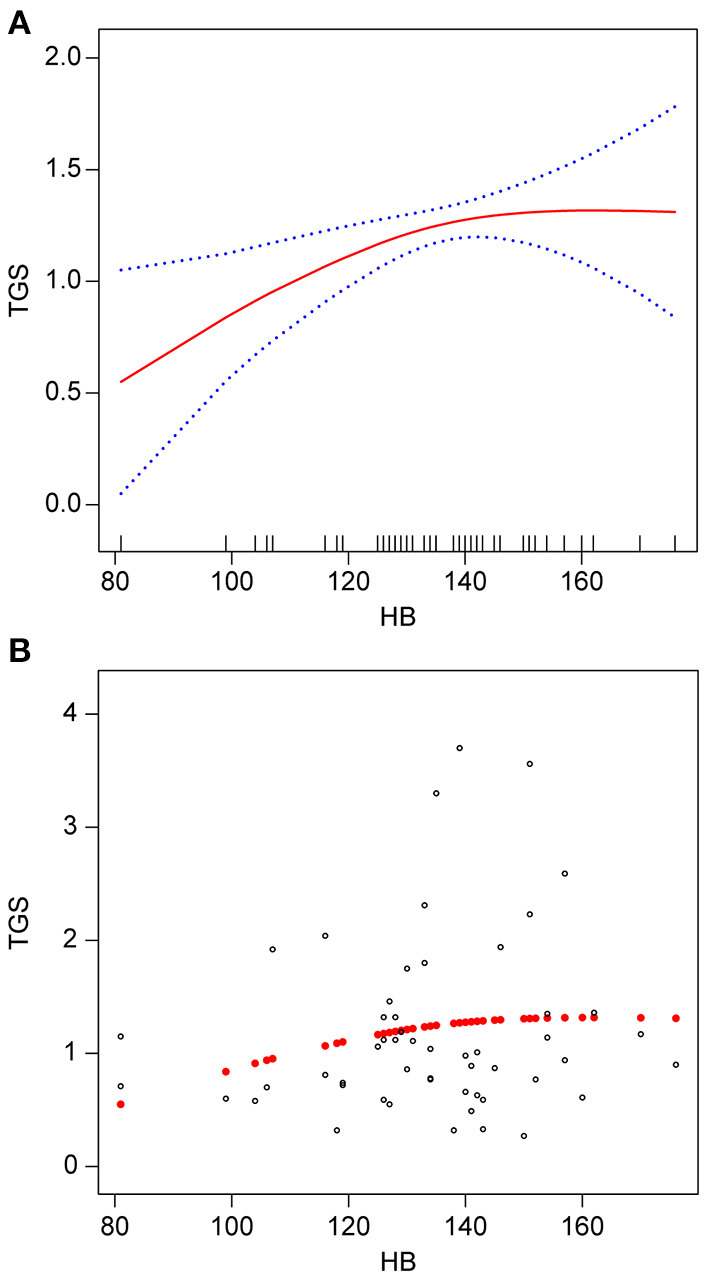
Association between Hb (g/L) and TGs (mmol/L) in patients with hemorrhagic MMD. **(A)** smooth fitted curve of Hb and TGs, **(B)** scatter plot for the distribution of Hb and TGs. The solid red line represents the smooth curve fit between the variables. The blue bands represent the 95% CI of the fit. The model was adjusted for age; smoking status; alcohol consumption; BMI; disease type; TC; HDL-C; LDL-C; VLDL-C; and lipoproteins.

**Figure 4:**
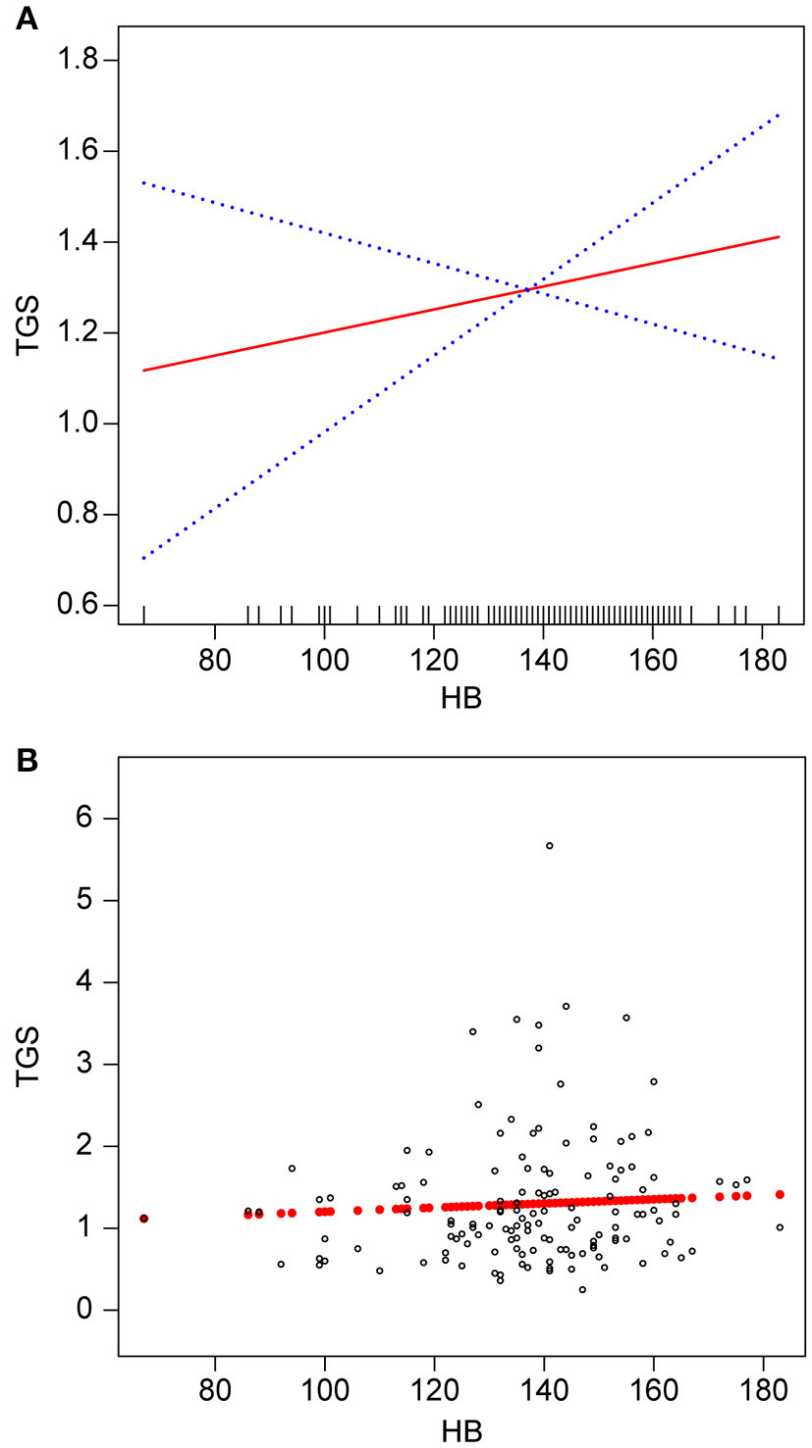
Association between Hb (g/L) and TGs (mmol/L) in patients with ischemic MMD. **(A)** smooth fitted curve for Hb and TGs, **(B)** scatter plot for the distribution of Hb and TGs. The solid red line represents the smooth curve fit. The blue bands represent the 95% CI of the fit. The model was adjusted for age; smoking status; alcohol consumption; BMI; disease type; TC; HDL-C; LDL-C; VLDL-C; and lipoproteins.

Smooth fitted curves were also plotted separately for patients with hemorrhagic MMD and those with ischemic MMD (Figures 3, 4, respectively). Both subgroups exhibited a positive relationship between the TG and Hb concentrations.

## Discussion

We observed a non-linear positive correlation between Hb and TG levels in patients with MMD. Further sensitivity analysis suggested a critical positive relationship between Hb and TG levels. Up to an Hb concentration of 141 g/L, we discovered that the TG concentration increased with the Hb concentration. However, above that threshold, there was no association between these two parameters. Upon stratified analysis of patients with hemorrhagic MMD and those with ischemic MMD, the Hb concentration was positively associated with the TG concentration in both groups.

MMD is an uncommon cerebrovascular illness marked by the creation of an aberrant network of collateral vessels, often leading to ischemic and hemorrhagic strokes (1, 2). The Hb level is an easily accessible and sensitive clinical indicator that reflects the physiological status of the body (7, 13). A recent study (12) showed a U-shaped association between hemoglobin concentration and stroke sequelae and recurrence, with either too high or too low hemoglobin concentrations being associated with stroke disability, death and recurrence. Meanwhile, a multicentre study (14) noted that elevated hemoglobin concentrations within 3 months of onset were associated with poor prognosis in men but not significantly in women with cerebral hemorrhage. Several previous publications (15–17) suggest that dyslipidaemia is a known risk factor for cerebrovascular disease. A high concentration of TGs is an independent risk factor for ICA stenosis, which is strongly linked to the development of MMD (5). A potential link between the pathophysiology of MMD and aberrant lipid metabolism has recently been demonstrated (18). Our team found a positive association between SUA and TG in a previous study (6) and concluded that early prevention of dyslipidemia could help reduce the incidence of cerebrovascular disease. Our data demonstrated that the Hb and TG concentrations of Chinese patients diagnosed with MMD were positively related after adjusting for other factors. These findings suggest a potential overlapping mechanism between Hb concentration and abnormal lipid metabolism.

Different derivatives of Hb are formed through oxidation, each exerting different pro-oxidative and pro-inflammatory effects, which can increase the sensitivity of vascular endothelial cells to oxidant-mediated injury and cause lipid peroxidation through the release of heme and redox-active iron, thereby leading to inflammation of the vascular wall (19, 20). Several researchers (21, 22) consider oxidative derivatives formed by Hb important causes of cerebrovascular disease. Those conclusions are largely in line with what we discovered.

To the best of our knowledge, there is limited information on the relationship between lipid metabolism and Hb in patients with MMD. We are also not aware of previous studies on the relationship between Hb and TGs in individuals newly diagnosed with MMD. Future MMD predictive models may benefit from our results, possibly leading to the development of a clinically accessible indicator for MMD diagnosis.

Our study had several strengths. First, we explored the non-linearity of the relationship between the two primary parameters. Second, as this was an observational study, we employed rigorous statistical adjustments to minimize the effects of influencing factors.

This study also had certain limitations. First, our study population comprised patients newly diagnosed with MMD in Southwest China, and we excluded certain categories of patients, which may limit the generalizability of our findings. Second, family history is a significant feature of patients with MMD, although only a few family members with MMD were discovered in the data we collected.

In summary, we revealed in the current study a link between Hb and TGs in patients recently diagnosed with MMD; this link may be related to the development of MMD.

## Data availability statement

The original contributions presented in the study are included in the article/Supplementary material, further inquiries can be directed to the corresponding author.

## Ethics statement

This study was approved by the Affiliated Hospital of Jining Medical University Institutional Review Board (approval number: 2021C107). Written informed consent for participation was not required for this study in accordance with the national legislation and the institutional requirements.

## Author contributions

GL contributed to conception and formal analysis. YS contributed to data curation, resources, and writing the original draft. FJ contributed to funding acquisition, project administration, validation, and reviewing and editing the original draft. HZ contributed to investigation. SF contributed to methodology. JL contributed to software. YL contributed to supervision. CC contributed to visualization. All authors contributed to the article and approved the submitted version.

## Funding

This study was supported by Research Fund for Lin He's Academician Workstation of New Medicine and Clinical Translation in Jining Medical University, JYHL2018FMS16, and the Key Research and Development Program of Jining Science and Technology (2018SMNS005), a Project of Shandong Province Medical Health and Technology Development Program (2014WS0518). The funders had no role in the study design; in the collection, analysis or interpretation of the data; in the writing of the report; or in the decision to submit the paper for publication.

## Conflict of interest

The authors declare that the research was conducted in the absence of any commercial or financial relationships that could be construed as a potential conflict of interest.

## Publisher's note

All claims expressed in this article are solely those of the authors and do not necessarily represent those of their affiliated organizations, or those of the publisher, the editors and the reviewers. Any product that may be evaluated in this article, or claim that may be made by its manufacturer, is not guaranteed or endorsed by the publisher.
