# Supplementary material for: The relationship between hemoglobin and triglycerides in moyamoya disease: A cross-sectional study
Source: Front Neurol. 2022 Sep 8;13:994341. doi: 10.3389/fneur.2022.994341 (PMC9493253; doi:10.3389/fneur.2022.994341)
Supplement: Supplementary file 1 [file Data_Sheet_1.pdf]

Supplementary Material

1 Statistical analysis

Continuous variables are presented in two ways: normally distributed continuous variables are presented as mean ± standard deviation, while the non-normal variables are reported as median. Categorical variables were described as frequencies or percentages. $\chi 2$ test for categorical variables, the one-way ANOVA test for normally distributed data, or the Kruskal-Wallis test for non-normally distributed data was performed to test the differences between Hb concentration groups (divided into quartiles). The entire process of data analysis can be divided into two steps. Step 1: Adjusted models were constructed based on Table 1 and 2 using univariate and multivariate linear regression. Step 2: A generalized additive model and smoothed-curve fitting (penalized spline smoothing) were used to address the nonlinearity of the Hb and TGs data. When nonlinearity was detected, the inflexion point was first calculated using a recursive algorithm followed by constructing a two-piece linear regression model on either side of the inflexion point. The best-fit model was determined based on the P-value of the log-likelihood ratio test. A sensitivity analysis was performed to ensure the robustness of the data analysis. We converted Hb levels to a categorical variable and calculated the P-value for trend, in order to validate the results for Hb levels as a continuous variable and to monitor the possibility of nonlinearity. All analyses were performed using the R statistical packages (http://www.R-project.org, R Foundation) and EmpowerStats (http://www.empowerstats.com, X&Y Solutions, Inc., Boston, MA). P-values <0.05 (two-sided) were considered statistically significant.
